# Successful Men

**Published:** 1876-02

**Authors:** 


### SUCCESSFUL MEN.

Who are they? They are those who, when boys, were compelled to work, either to help themselves or their parents; and who, when a little older, were under the stern necessity of doing more than their legitimate share of labor; who as young men had their wits sharpened by having to devise ways and means of making their time more available than it would have been under ordinary circumstances. Hence, in reading the lives of men who have greatly distinguished themselves, we find their whole youth passed in self-denials, of food, and rest, and sleep, and recreation. They sat up late, and rose early to the performance of imperative duties; doing by day-light the work of one man, and by night the work of another.

Said a gentleman, the other day, now a private banker of high integrity, and whom we knew had started in life without a dollar: "For years together I was in my place of business at sunrise, and often did not leave it for fifteen and eighteen hours."

Let not, therefore, any youth be discouraged if he has to make his own living, or even to support besides a widowed mother, or sick sister, or unfortunate relation, for this has been the road to eminence of many a proud name. This is the path which printers and teachers have often trod: thorny enough at times, at others so beset with obstacles as to be almost impassable; but the way has cleared, sunshine came, success followed, then the glory and renown!

A young man writes us: "I am an humble school-teacher; with the duties belonging to half a hundred pupils, I issue a monthly, printed nine miles away, and do all the folding, stitching, binding, and mailing of three thousand copies, with a deep feeling that good may be done. I hope I will succeed."

Certainly he will succeed! For he has the two great elements of success: a will to work, and a heart in the right place; a heart whose object is not glory, but good.

But too often has it happened that there comes in, between the manly effort and a glorious fruition, disease, crippling the body, depressing the mind, and wasting and wearing away the whole man. Who does not remember grand intellects which have gone down in the night of a premature grave? Who has not seen young men with magnificent minds, standing on the borders, looking wistfully, oh! how wistfully! over, but unable to "go in and possess the land" only for the want of bodily health? A health by no means wanting originally, but sacrificed; pitilessly, remorselessly sacrificed by inattention and sheer ignorance; learned in every thing else; critically informed in every thing else; perfect masters of every thing else, except the knowledge of a few general principles as to the care of the body; principles which could be perfectly mastered in any twenty-four hours by a mind accustomed to think.

Within a few months two men have died in the very prime and vigor of mental manhood, being not far from fifty, one the first scholar of his time; the other, one of the very best and most useful men of the age; both of them the victims of wrong habits of life; habits framed in youth, and utterly repugnant to the commonest dictates of common-sense. Some of the most useful rules for the preservation of the health of the young, while obtaining an education, are these:

1. Keep the feet always dry and warm.

2. Eat thrice a day, at regular times; not an atom between meals; taking for supper only a piece of cold bread and butter with a single cup of any warm drink.

3. Go to bed not later than ten o'clock, and never remain there longer than eight hours at farthest, not sleeping a moment in the day-time.

4. Cool off with the utmost slowness after all forms of exercise; never allowing an instant's exposure to the slightest draught of air while in a state of rest after that exercise.

5. If the bowels fail of acting daily, at the regular hour, eat not an atom until they do, but drink all that is desired, and give more time than usual to out-door exercise, for several days.

These five rules can easily be remembered, and we appeal to the educated physicians of all lands for confirmation of the truth of the sentiment, that a judicious habitual attention to them is essential to the preservation of sound health, and the maintenance of a good constitution the world over. Their proper observance would add a young lifetime to the average age of man.

